# Women's Preferences for Penis Size: A New Research Method Using Selection among 3D Models

**DOI:** 10.1371/journal.pone.0133079

**Published:** 2015-09-02

**Authors:** Nicole Prause, Jaymie Park, Shannon Leung, Geoffrey Miller

**Affiliations:** 1 Department of Psychiatry, University of California Los Angeles, Los Angeles, California, United States of America; 2 Department of Psychology, University of New Mexico; Albuquerque, New Mexico, United States of America; Knox College, UNITED STATES

## Abstract

Women’s preferences for penis size may affect men’s comfort with their own bodies and may have implications for sexual health. Studies of women’s penis size preferences typically have relied on their abstract ratings or selecting amongst 2D, flaccid images. This study used haptic stimuli to allow assessment of women’s size recall accuracy for the first time, as well as examine their preferences for erect penis sizes in different relationship contexts. Women (*N* = 75) selected amongst 33, 3D models. Women recalled model size accurately using this method, although they made more errors with respect to penis length than circumference. Women preferred a penis of slightly larger circumference and length for one-time (length = 6.4 inches/16.3 cm, circumference = 5.0 inches/12.7 cm) versus long-term (length = 6.3 inches/16.0 cm, circumference = 4.8 inches/12.2 cm) sexual partners. These first estimates of erect penis size preferences using 3D models suggest women accurately recall size and prefer penises only slightly larger than average.

## Introduction

Both men and women often have reported discomfort with the appearance of their genitals. While not as common of a concern as body weight, muscularity, amount of head hair and body hair, or height, penis size was a concern for 68.3% of 200 men in one study [1]. Concerns about genital appearance are unique compared to other concerns about physical appearance. First, only intimate partners generally know the appearance of genitals. In contrast to the penis, body weight, acne, and other features are easily observed, informing feelings of attraction early in interactions. While indicators of penis size include ethnicity [2] and finger length and ratio [3, 4], most proposed cues of penis size, including male height and foot size [5], weight [6, 7], shoe size [8], and age [9], are unreliable. Second, no diet, pill, or exercise regime affects the size or shape of genitals. However, about half of men in one study believed that they could change their penis size through non-surgical means [10]. Little can be done to change the appearance of the penis. Contrary to some public opinion, it also is worth noting that discomfort with the appearance of the penis is not impacted [11], or is positively impacted [12], by viewing sex films. Given that only intimate partner(s) view the penis, the appearance is relatively immutable, and sex films are not causing dissatisfaction, partner perceptions of the penis appearance seem to most likely to impact men’s feelings about the features of their penis.

The expectations that men have about women’s penis size preferences appear to drive anxiety and dissatisfaction more than some inborn dissatisfaction. In the first questionnaire to examine the nature of dissatisfaction with the penis directly, three of the ten items concerned a partner’s perception [13]. These included “I will be alone and without a partner” and “I will be laughed at by a partner in a sexual situation”. These anxieties may be unnecessary. For example, while men and women agreed that the “ideal” penis length was longer than what they thought was average, men mistakenly reported that women would find an even longer penis ideal than the women actually did [10]. Furthermore, most men seeking surgery to increase their penis size (e.g., [14, 15]), actually fall within the normal penis size range [16].

Concerns about penis size affect men’s sexual satisfaction and functioning. Of course, penis size need not affect sexual functions like orgasm, sexual drive, or pain experience. However, men who are less satisfied with their penis report more sexual health problems [17]. A smaller penis decreases sexual confidence [18], which may be why penis size is related to sexual function. Another reason penis size may be related to sexual functioning is that anxiety concerning the partner’s response may be calculated as a cost of the relationship, which leads him to experience broad sexual dissatisfaction [19].

The context of the sexual relationship could influence penis size preferences. For example, the goal of the sexual interaction with a one-night partner tends to be pleasure [20]. Women recognize that infection risks are higher from a one-night partner [21]. While women adjust their behaviors for this risk, being less likely to engage in anal sex [22] and more likely to use condoms [23] with one-night partners, such risky behaviors themselves are often experienced as pleasurable [24]. On the other hand, vaginal intercourse always causes tears in the vaginal mucosa [25] especially in the sensitive posterior fourchette [26], so women might prefer a smaller penis less likely to stress their physiology for regular, long-term mates. Thus, women might shift their preferences for penis size depending on the type and duration of sexual relationship.

Studies of penis size preference to date have relied on numerical size estimates, vague qualitative descriptions, or 2-D line drawings. For example, some studies have asked participants to specify penis length preferences in centimeters [27]. Another study asked participants to indicate their preference from reading erotic passages with three qualitative penis size options (small, medium, large) [28]. Yet, humans judge sizes most accurately when visual and haptic information are available together [29]. Both sources of data are usually available in sexual interactions. Thus, in this study, three-dimensional (3D) models were used with the hope of increasing accuracy, ecological validity, and external validity. Also, most studies of penis size preference have portrayed or asked about the penis in its flaccid state [30, 31]. This may be problematic, because the relationship between erect and flaccid sizes has been reported as negligible [32, 33] moderate (r = .44 in [34], r = .78 in [35]), and strong (rho = .77 in [6], r = .79 in [32]). It is unclear how well flaccid size reflects erect size. Of course, intercourse can occur only with a sufficiently rigid penis [36]. Thus, it seemed important to characterize preferences for penis size in its erect state. The current study used 3D models of erect phalluses to characterize women’s penis size preferences for the first time.

Three-dimensional (3D) printing is just beginning to be used to assess shape perception and categorization. On the one hand, visual 2D information as compared to haptic information (from 3D) result in similar solutions for object similarity [37]. Each mode of information (visual or haptic) also improves categorization in the other domain [38, 39]. 3D printing could allow representation of highly problem-specific, complex structures [39]. Haptic information from 3D objects improved shape identification compared to raised lines alone [40] and improves later performance in the visual domain [41], possibly by improving discriminability [42]. Also, haptic information is robust to differences in perceptual acuity, such as occur with aging [43], which make such stimuli attractive when the visual acuity of participants may vary. This study extends the existing work using 3D stimuli to assess size preferences. This approach also permitted characterization of women’s ability to accurately recall the size of erect phallus models for the first time.

When flaccid and “stretched” penis sizes are characterized [44], largely by self-measurement [45], they predict erect size surprisingly poorly. Yet there are relatively few studies of erect penis size. This may reflect cultural taboos against researchers or doctors interacting with men who are in a sexually aroused state. One study had men judge their own erect size in relation to a banknote’s length [46]. Two studies of erect penis sizes provided kits for home measurement [47, 48]. Such self-measurements of length and circumference show fairly good test-retest reliability (r = .68 to .90, [47]). Pharmacologically-induced, physician-measured erections identified an average length of 12.89 cm (SD = 2.91) and circumference of 12.3 cm (SD = 2.9; [32]). These were somewhat shorter in length (*M* = 14.15, *SD* = 2.7), yet similar in circumference (*M* = 12.23, *SD* = 2.2), compared to a recent, large survey [48].

Women’s penis preferences may vary with their relationship expectations. Women prefer more masculine partners for shorter-term sexual relationships [20]. Women also value intelligence more, and attractiveness less, for long term, as compared to short term, partners [49]. More masculine traits, such as lower voice pitch [50] and (to some extent) larger penis size [51, 52] are correlated with testosterone levels, which also may influence men’s mating goals and attractiveness. Since a larger penis size is perceived as more masculine [53, 54], we predict women will prefer a larger penis for shorter-term sexual relationships.

Women likely make penis size judgments partly using their recalled experiences. Yet, it is unclear how accurately women can recall penis size. Exposed to nude male images, women do attend to the genital area [55, 56]. People can generally recall if a penis was described as “large”, “medium”, or “small”, or not described at all [28]. In the current study, women’s ability to recall penis size was tested by match-to-sample recall, both immediately and after a delay of ten minutes.

## Materials and Methods

### Stimuli: The penis models

**Fig 1:**
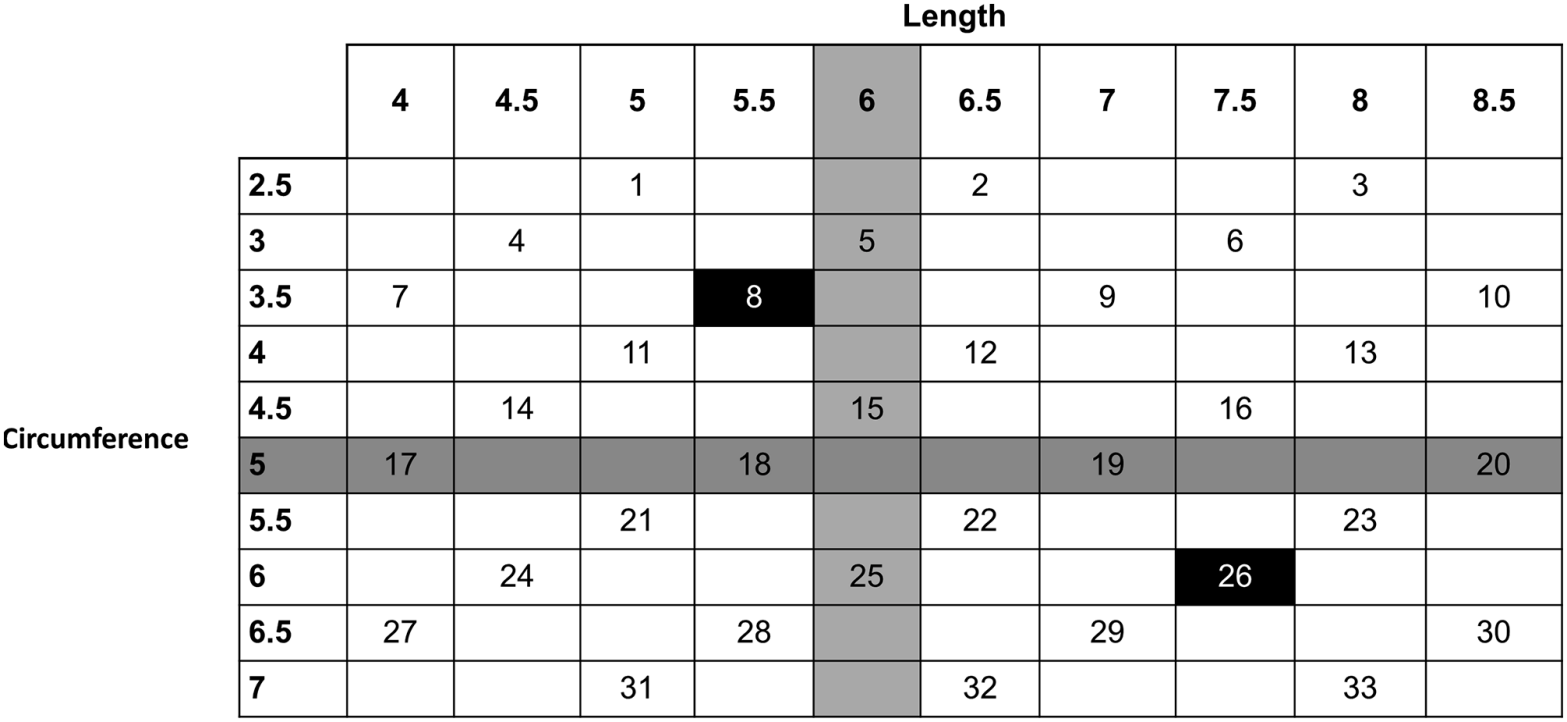
Sizes of printed models. Shading indicates the average penis length and girth in the USA. Bold indicates models used for recall (immediate/delayed, counterbalanced) tests. Units are in inches.

Based on previous studies (see above) about the distributions of penis length and circumference, the average American erect penis length was estimated as 6 inches (15.2 cm) and circumference as 5 inches (12.7 cm). Models were created to range +/- 3.0 S.D. across each dimension (see Fig 1). This resulted in length ranging 4.0 inches to 8.5 inches (10.2 cm to 21.6 cm), and circumference (circumference) ranging from 2.5 inches to 7.0 inches (6.4 cm to 17.7 cm), using 0.5-inch (1.3 cm) increments (see Fig 1). This yielded a 10 X 10 matrix of 100 possible sizes. However, such a large choice set could overwhelm participants. We chose to sample 1/3 of this space, yielding 33 models across the range of space.

**Fig 2:**
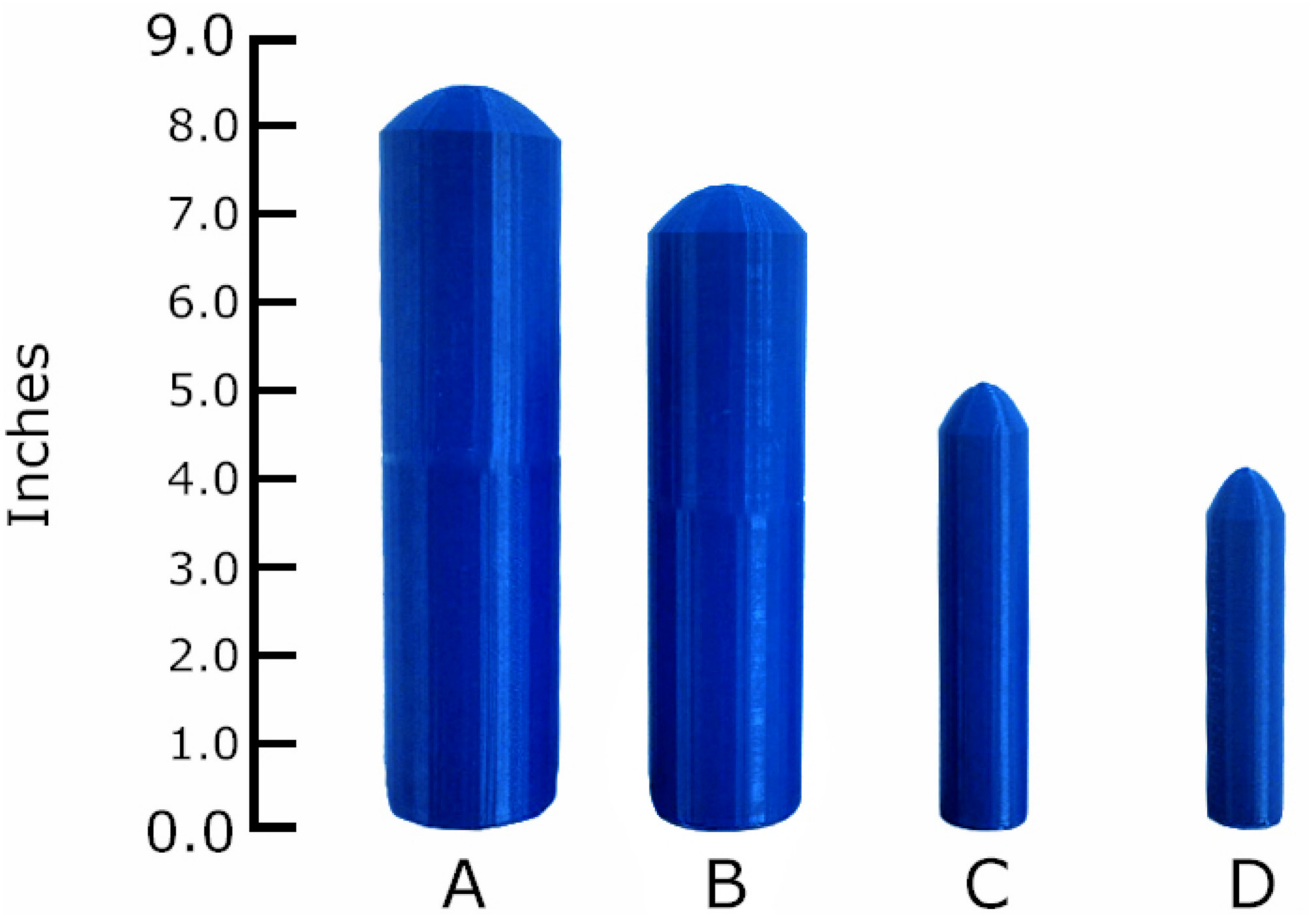
Penile Models. A) Computer graphic representation of one of the ‘print files’ used to produce the 3D penis models. B) Examples of (4 of 33) 3D models showing length in inches. A and D represent the largest and smallest models in the set, respectively; B and C represent the two models (counterbalanced) used to test recall for size.

The penis model shape was a cylinder, representing the shaft, topped by a dome, representing the penis head (see Fig 2). Of course, the human penis shaft is comprised of three corpora that could be better represented by a rounded triangle and a more complex glans. Also, no veins, testicles, or other details of the penis were portrayed. These details were not represented for three reasons. First, there are no mathematical descriptions available to accurately represent normal proportions of more complex penile structure. Second, women generally rate male nudes as less attractive than heterosexual men rate female nudes [57], so making the penis model more realistic might have provoked negative responses. Third, the study was focused on overall penis size, not penis shape or surface details. While one motivation behind the current study was to improve the ecological validity of the stimuli, these concerns suggested starting with a more simplistic, erect penis model.

Dimensions of commercial penile models do not vary systematically, so they were not appropriate for research purposes. Thus, the penis models were printed using a Makerbot Replicator 2 in blue ABS plastic (“Navy wool”; printer files for replications are at http://www.thingiverse.com/thing:518401). Files were created using object-oriented Tinkercad [58] and compiled to.stl formats in G-Replicator [59]. Models were light-weight, sturdy plastic with a smooth surface (see Fig 1). After printing, models were checked by measuring tape to ensure accuracy of length and circumference. None required reprinting for accuracy. The models were identified by randomly assigned letters (e.g., “M”, “CC”) written on the bottom of each. This was done to reduce the influence of “largest” and “smallest” anchors and also to eliminate the need for women to measure or infer specific size. The 33 models were evenly split (11, 11, 11) in a three-tier wire basket to ease women’s ability to find the desired model. Baskets were randomly shuffled between participants to reduce selection bias.

### Participants

Volunteer were recruited by flyers around the California university campus, the neighborhood, and local physicians’ offices. The flyers stated that women were requested to volunteer for a study concerning sexuality. The flyers also stated that participants must be female, at least 18 years old, sexually attracted to men, and would be paid $20. The flyer did not mention penis size preferences. Women volunteered by either phone or an online form requesting a phone call. They completed a phone screening to confirm their eligibility (e.g. being aged 18 or over, being sexually attracted to men) before being scheduled as participants.

### Procedures

Upon a participant’s arrival, the Informed Consent document was provided, and women were given time to study it. Afterwards, they were given a chance to ask questions, then the experimenter asked whether they still wish to participate. If the participant verbally consented, the experimental protocol started. The Informed Consent document stated that continuing at this stage constituted consent. Participants never provided their names. Informed Consent was not documented using identifiable personal information, because it was unclear whether the new procedures might influence participants’ willingness to report their penis size preferences.

Next, the participant answered questionnaires (described below) presented on a computer in a private room, using a secure connection, on private laboratory server space scripted by the first author in php5. This took about 50 minutes and included the penis size preference tasks and questionnaires (see below). Computer presentation of questionnaires has been shown to increase the reporting of socially less desirable behaviors [60]. After the questionnaires, she completed a 10-minute computer task (data to be reported elsewhere) assessing attention to sexual images. Afterwards, the participants was debriefed, offered the opportunity to ask questions, and given $20 cash. The study protocol, including Informed Consent protocol, was approved by the University of California, Institutional Review Board.

### Questionnaire

The self-report questionnaires included demographic information (e.g., age, ethnicity, sexual orientation), sexual history (e.g., number of sexual partners, sexual coercion, whether penis size played a role in relationship dissolution(s), etc.), and current sexual functioning (e.g. orgasm rates, ease of lubrication, relationship monogamy status, pain during intercourse). These were used to characterize the sample. Other personality questionnaires were included, such as the Sexual Desire Inventory [61] and the Sociosexual Orientation Scale [62] to characterize the sample.

### Size preference and recall: Recall accuracy

After completing the other questionnaires, the experimenter entered with one of the two test models. Two of the original 33 models were randomly selected and reprinted (indicated in black cells in Fig 2). The experimenter informed the participant that she would be handed a model. She was instructed that she would be asked to try to recall the size of the model after inspecting it. During the inspection, she was asked not to measure the model using any objects in the room, but no instruction was provided regarding how she used her own hands. Then, the experimenter left for 30 seconds (without observing the participant’s inspection process), returned, took the test model from the participant and out of the testing room, and asked the participant to select which penis model (from the 33 described above) was most similar in size to the test model she just handled. The participant recorded the letter code from the bottom of that model into the computer.

The delayed-recall task was similar, except this time, the participant did not immediately search for the model. Instead, she was given ten minutes to complete the penis size preference questionnaire (below). The preference questionnaire would increase memory interference, which is desirable for ecological validity as women asked to recall a former partner’s penis size may have sex with other new partners in the delay. After this, the participant was instructed to attempt to locate the second model (from the 33 described above). The test models were counter-balanced, so the recall type (immediate or delayed) would not be confounded with test model size (larger or smaller).

### Size preference and recall: Penis Size Preferences

After completing the immediate recall task, participants answered 15 questions about their penis size preferences. Each involved picking one penis size model from amongst the 33 models available. The option “No answer” also was available for each. For this study, the key questions were to select the model that they believed best reflected the average of men, which size is most likely to carry a sexually transmitted infection, and which size she would prefer for different expected relationship durations. The questions about preferences for different types of partners were a bit more complex. For one-time partners the question was:

“Imagine you're single and you're out at a restaurant with some friends. You meet an attractive man who is also single. He seems kind, intelligent, funny, and has a great job. You are feeling sexually aroused. He says he's in town for a conference but he has to fly back home tomorrow afternoon. If you could spend only this one night with him, what size would you want him to be?”

For long-term partners the question was: “What would be the ideal size for a husband or serious, long-term boyfriend?” The question regarding shorter-term partners clearly included much more detail. This was done in an attempt to control for intervening variables not of interest. For example, if a woman doubted at all for her safety with an unknown partner, she might select smaller models in the event of sexual assault. Thus, safety cues were included in the characterization.

### Data analyses

Recall error was calculated as the difference of the dimension the participant chose minus the size of the actual sample. Thus, a positive number would indicate that participants chose a model larger than what they were shown. A within-participant ANOVA was calculated with the interaction of dimension (length, circumference) by recall (immediate, delayed. Put another way, the accuracy of recall could be affected by length or circumference being recalled better than the other dimension (dimension factor), by the length of the delay was until they selected a model (recall), or an interaction where length or circumference were recalled better at either the shorter or longer delay.

Descriptive data are provided regarding the size that women believed was average and the range women indicated for their “smallest” and “largest” sexual partner. To test whether women’s preferences differ by partner type, an ANOVA with dimension (length, circumference) X partner (one-time, long term) predicting preferred inches was conducted. A custom model was specified without dimension as a main effect, because dimensions were stipulated to be different in the generation of the stimuli.

## Results

### Participant demographics and sexual experience

**Fig 3:**
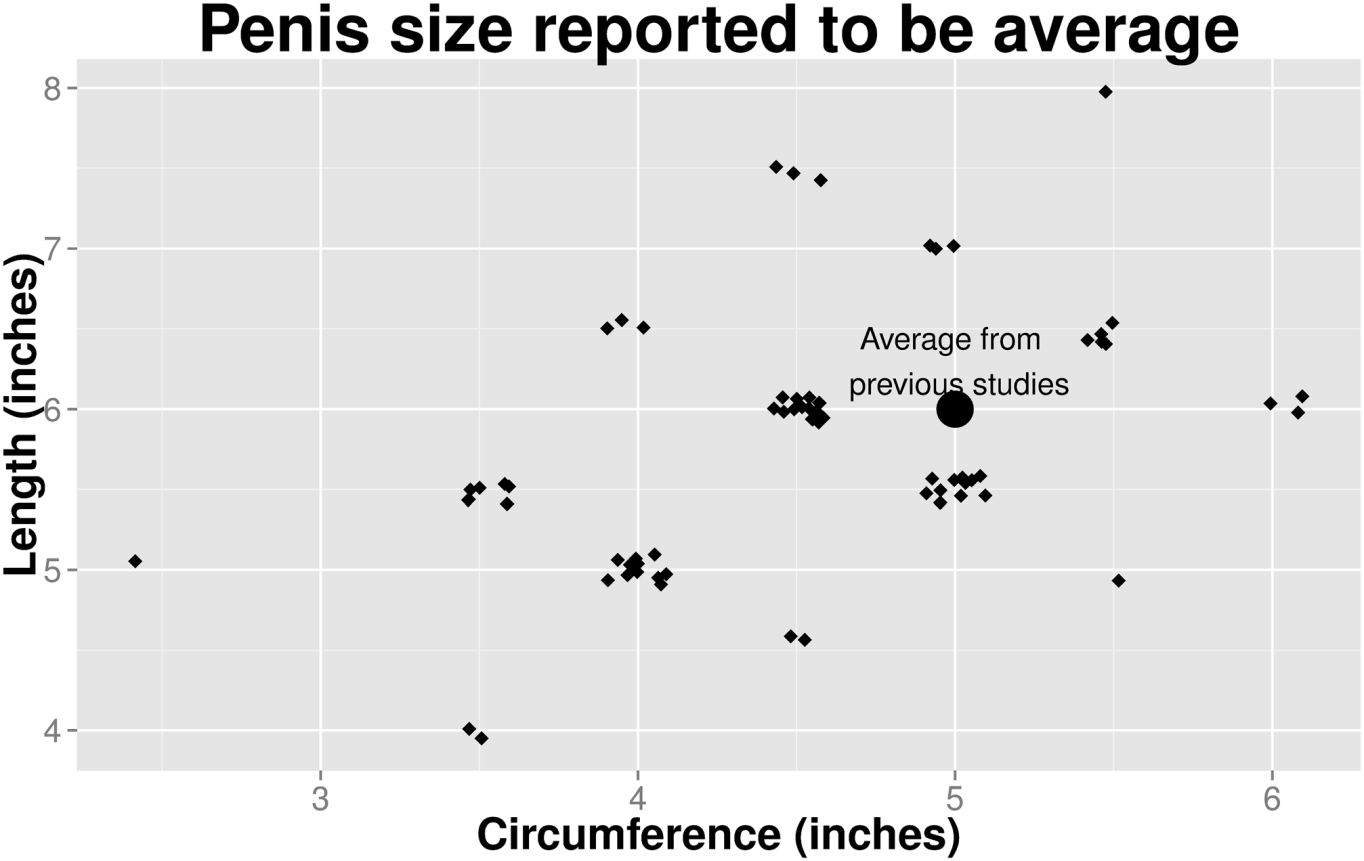
Size of model selected by women indicating the “average” penis size. (*N* = 75, *r* = .48).

**Fig 4:**
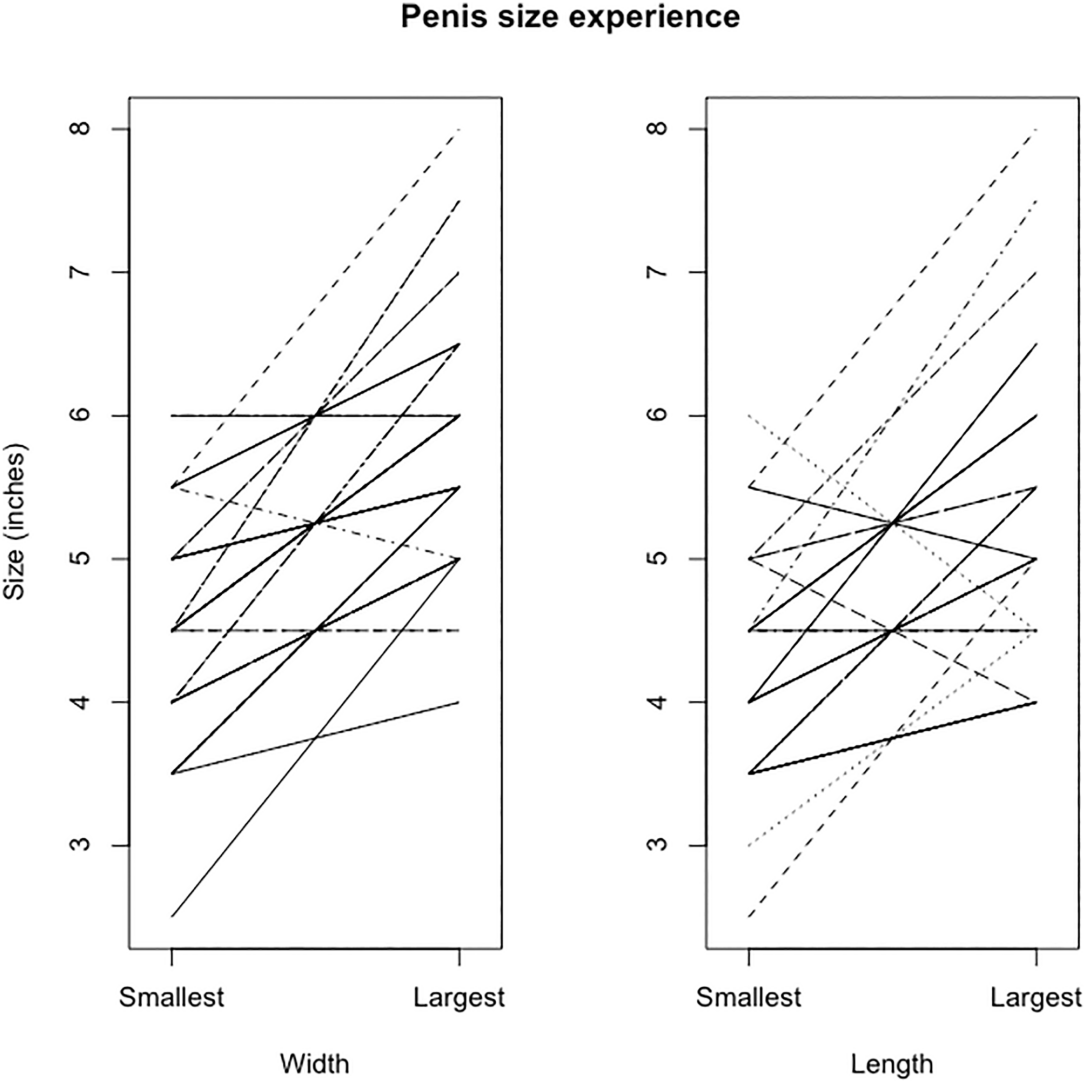
Largest and smallest penis experiences. No evidence of ceiling or floor effects in women’s choices indicating their largest and smallest sexual partner’s penis size.

All participants (*N* = 75) were screened to report sexual attraction to men, and ranged in age from 18 to 65. They were California residents, mostly white or Asian, sexually experienced, currently in a sexual relationship, and had sex recently (see Table 1). Twenty-seven percent of women reported that they had ended a relationship due, in part, to a mismatch between their penis size preference and their partner’s penis size (see Table 1). More women cited that the penis was too small as a problem, rather than that the penis was too large. The length and circumference of the model that each woman believed best represented the “average” penis size is presented in Figs 3 and 4 shows every woman’s selection of the “smallest” and “largest” sexual partner with whom she had contact.

**Table 1:** Demographic characteristics of participants.

Variable	M	SD
Age	24.7	10.5
Intercourse partners (last 12 months)	3.2	5.3
Intercourse partners (in lifetime)	6.0	9.0
Number of penises touched (lifetime)	6.8	9.0
	**N** ^a^	**%**
Sexual orientation (self-identified)		
Heterosexual	36	57.1
Bisexual	10	15.9
Lesbian^b^	8	12.7
Asexual	6	9.5
Queer	3	4.8
Did not identify	11	14.7
Race^c^		
White	28	37.3
Asian	24	32.0
Hispanic (non-white)	16	21.3
Black	10	13.3
Pain with intercourse		
None	28	37.3
Mild	20	26.7
Discomforting to excruciating	27	36.0
Frequency of intercourse (last month)		
Not once	26	35.1
1 to 3 times a month	22	29.3
About once a week	10	13.5
2 or 3 times a week	13	17.6
4 times a week or more	3	4.0
One night stand experience (lifetime)		
Not once	34	45.3
Once or more	41	54.7
Penis size concern^d^		
A lot more	0	0
A little more	11	15
About the same as other women	37	49
A little less	13	18
A lot less	12	16
Relationship ended due to penis size preference^e^		
Penis too large	5	7
Penis too small	15	21

^a^ Numbers may not sum to total due to non-response.;

^b^ Recall that participants were required to report attraction to men to participate, thus a “Homosexual/Lesbian” self-identity did not preclude attraction to men;

^c^ Participants were allowed to indicate more than one option. Top 4 endorsed races or ethnicities are included.

^d^ Question wording “How much do you think you care about penis size compared to other women?”

^e^ Question wording “Have you ever stopped seeing a man because, among other reasons, his penis was too large[small] compared to what you wanted?”, number indicates count endorsing.

### Recall accuracy

**Fig 5:**
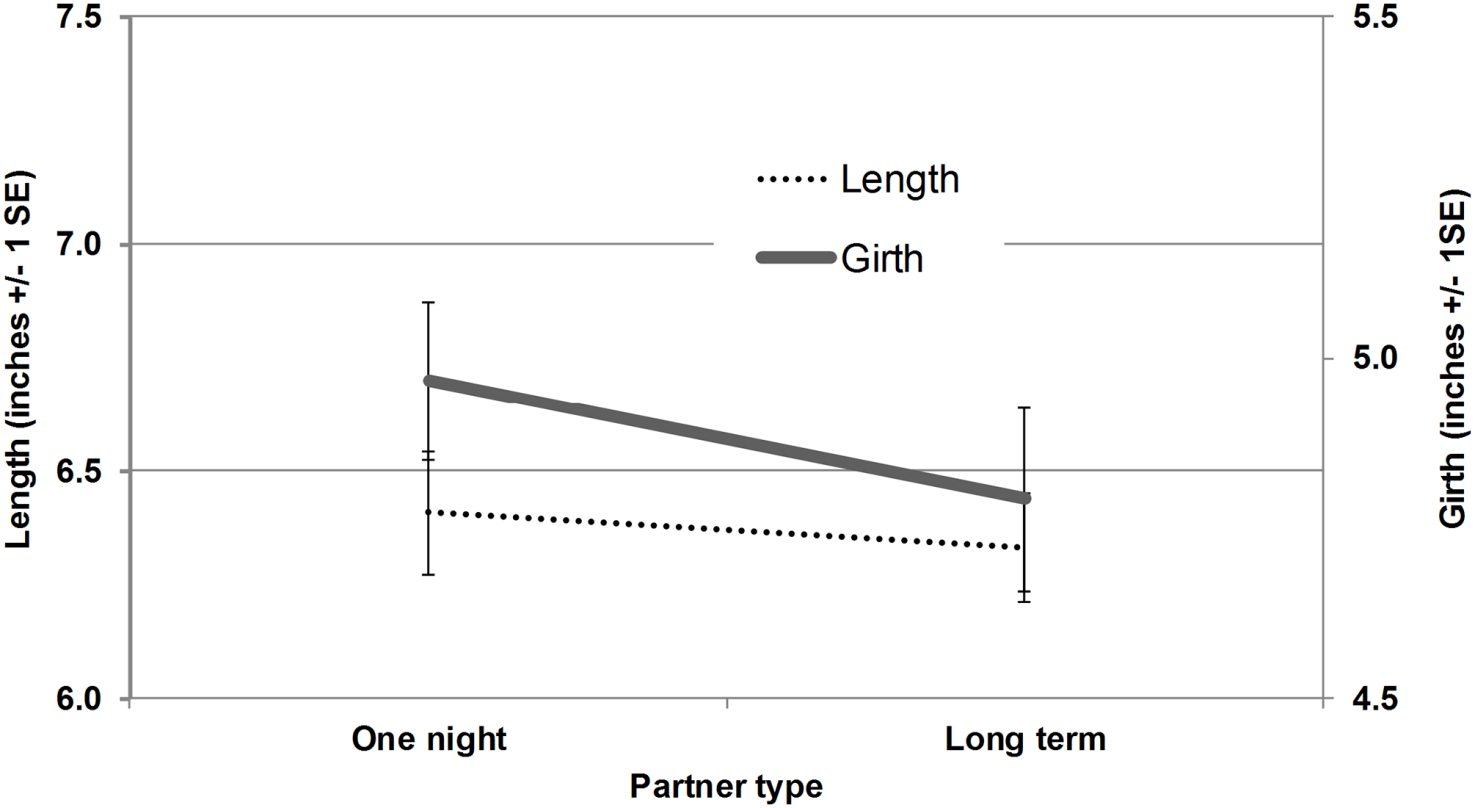
Recalled sizes (immediate and delayed) slightly shorter than actual model with most picking exact model. Note: “0” indicates the exact correct model was chosen. Positive values indicate that the selected model was larger than the target model.

Most (*N* = 48) women selected the exactly correct model (in both length and circumference) at immediate recall (see Fig 5). About half (*N* = 31*)* of women selected exactly the correct model at delayed recall. There was a main effect of dimension predicting model selection error (*F*(1,73) = 11.6, *p* < .001, η_p_^2^ = .14): participants slightly underestimated penis length after the recall interval (*M* = -0.18 inches or -0.46 cm error), but were very accurate recalling penis circumference (*M* = 0.02 inches or 0.05 cm error). There was no main effect of delay nor dimension X delay interaction despite high power (*f* = .1, *r* = .9, 1-β = .97). Given the high accuracy, analyses for preferences were conducted as planned.

### Does the expected relationship duration affect penis size preference?

**Fig 6:**
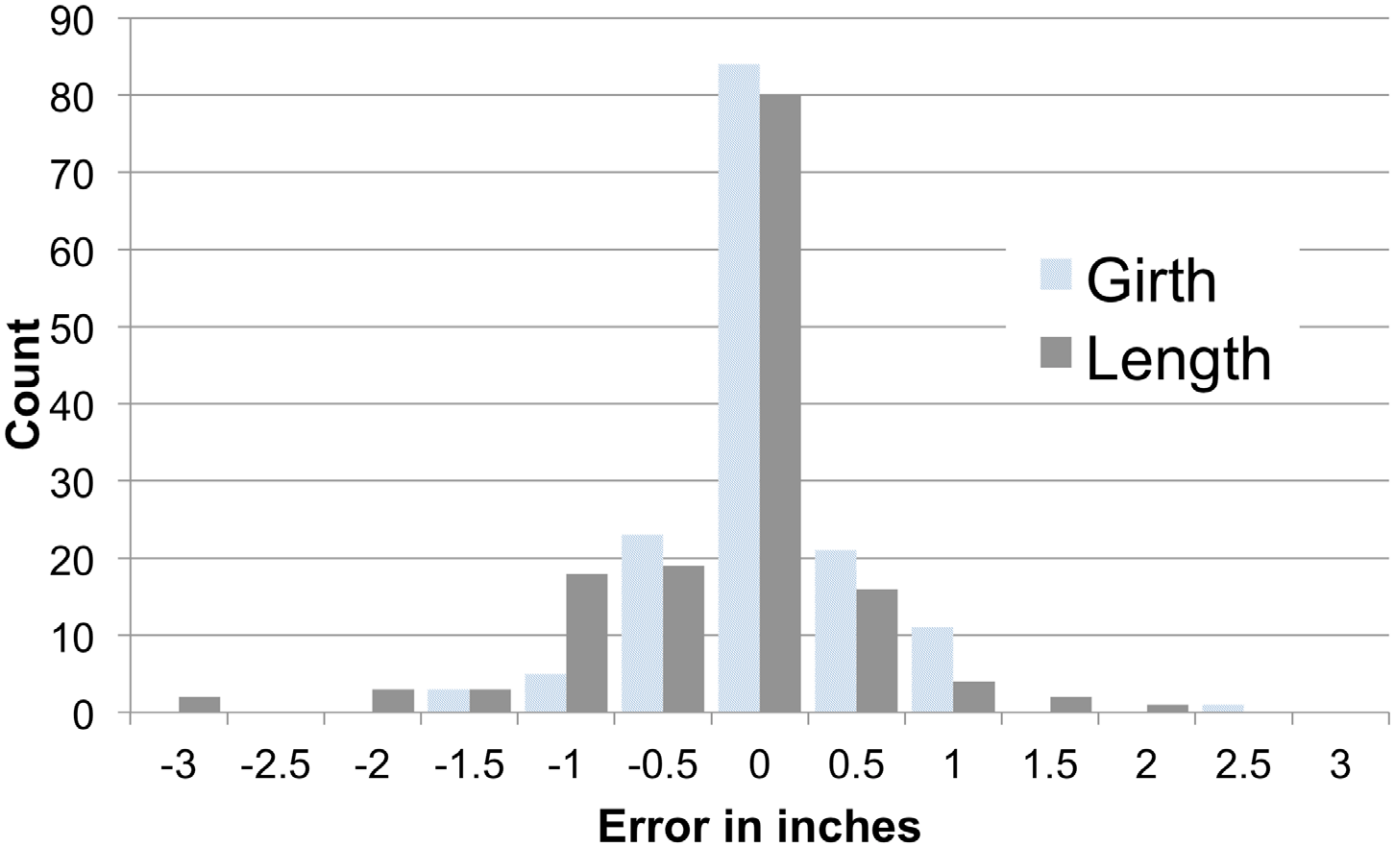
Preference for larger penis in one-time relative to long-term relationships.

For the penis size preferences for one-time or long-term partners, 15 women indicated “No answer”. Analyses were conducted on the remaining participants (*N* = 60). There was a small main effect for expected relationship duration, *F*(1,59) = 4.4, *p* = .04, η_p_^2^ = .07 (see Fig 6), such that participants preferred a slightly larger penis size in one-time (length = 6.4 inches or 16.3 cm, circumference = 5.0 inches or 12.7 cm) partners as compared to long-term partners (length = 6.3 inches or 16.0 cm, circumference = 4.8 inches or 12.2 cm). There was no interaction of dimension (length, circumference) and relationship duration. Using independent t-tests separately predicting length and circumference preferences for partner type resulted in a significant difference for the test of circumference (*t*(59) = 2.4, *p* = .02, *d* = .2) only. Women preferred a larger circumference in one-time partners (*M(SD)* = 5.0(.1)) relative to long-term partners (*M(SD)* = 4.8(.1)). As ANOVA corrects for multiple comparisons, it is a more appropriate statistical test for these data. These t-tests are noted for full disclosure of the analyses conducted. Only 16 women selected a model as “most likely to have an STI”, whereas most women declined to select a model. Of the women who did respond, the model selected as most likely to have an STI was significantly larger (*M(SD)* = 6.2(.3)) than the model women used to indicate their one-night stand (*M(SD)* = 5.8(.2)) preference, *F*(1,15) =, *p* = .01, η_p_^2^ = .35. This finding did not vary by the dimension (length, circumference).

## Discussion

Women attended one session in the laboratory during which they completed questionnaires about their sexual history and selected among 3D erect penis models to indicate their size preferences for one-time or longer-term partners. The state-space appeared to well-characterize the range of women’s experience, as their “largest” and “smallest” partners did not show evidence of ceiling or floor effects. Women tended to recall the size of the 3D models very well, only underestimating penis length. Women preferred a larger penis size (especially a larger circumference) for one-time partners as compared to long-term partners. While this preference for a larger phallus is above the average penis size, it is only very slightly above the average. While most declined to identify a penis size most likely to carry an STD, women selected even larger phallus sizes as the most likely to be infected with an STD.

A delay in model recall did not significantly worsen participant’s recall of the model size. In fact, women were generally very accurate in identifying the same model at both immediate and delayed recall. When they did make errors, they slightly underestimated model length. One possible explanation is that women care more about circumference, so they may attend to it more [63]. Some authors have argued that penis length actually is more important and “healthy” to desire than circumference (e.g., [64, 65]), but others have not replicated this reported pattern.

These data are generally consistent with Mautz et al. (2013), which asked women to rate the attractiveness of life-sized, projected, rotating drawings of male figures with flaccid penises of various sizes. Their participants preferred phalluses 2SD above their estimated population-average penis size, whereas our participants preferred penises that were only a little above average. This difference may be due to their images depicting flaccid penises, whereas our models depicted erect penises.

Since women’s preferences for both relationship types were slightly larger than the average male, the preferred size for the one-time partner was farther from the average. Novelty itself contributes to pleasure [66], so seeking a more novel-sized penis may be consistent with a goal to pursue pleasure primarily in one-time partners. Women may prefer a smaller penis size in a long-term partner compared to a one-time partner for reasons of both physical comfort and a preference for less masculinity in a longer term partner [67]. The difference in pleasure motive is also suggested by genital physiology. A larger circumference might stretch the vaginal opening such that the deep structures (clitoral crura and vestibular bulbs) are more stimulated, and the clitoral glans is more stimulated by penis movement [68]. Also, the vagina is densely packed with pressure-sensitive mechanoreceptors that detect stretch sensations [69]. These appear finely tuned to detect variability in circumference, whereas the vagina is less sensitive to differences in other stimuli such as vibration or warmth [70]. Other studies also found that women prefer a relatively larger penis proportional to body size [31], especially with respect to circumference (e.g., [54]). Given that women typically experience more pleasurable and orgasmic sex in longer-term relationships [71], they might prefer a larger penis for short-term sex partly so the increased physical sensation compensates for the reduced psychological connection. In one notable exception, a preference for general body somatotype did not differ by the relationship duration [brief uncommitted versus long-term partners in 72].

A larger penis could contribute to infection risks, such that a larger penis on more risky one-time partners elevates risk. A larger penis has been associated with higher infection rates amongst men who have sex with men [73]. Also, an increase in friction during intercourse from a condom is associated with the introduction of more bacteria into the vagina [74, 75] and more vulvar erythema [74]. Finally, women report that condoms increase their experience of pain during intercourse [76, 77]. Anything that increases friction during intercourse may promote genital injury, indirectly increasing infection risk. A larger phallus would increase friction relative to a smaller phallus. These potential complications of a larger penis suggest why the human penis has not evolved to be larger.

Individual differences among the women were not examined in relationship to their penis size preferences, although various female traits could interact with their sexual health risks. For example, women with wider hips tend to have a higher proportion of one-time sexual partners [78]. While women’s vaginal depth and pelvic muscle tonicity has been characterized [79, 80], these traits have never been related to women’s penis size preferences. Presumably, given the variability in vaginal size and tonicity, some women would experience more tearing with a larger phallus than other depending on the morphology of their particular vagina.

Generating haptic stimuli was relatively cost-effective and simple. Free software was available for generating print files. Also, the print files are shared online to allow exact future replications. Undergraduate research assistants were able to create and monitor the work flow. The 3D printer used is now widely, cheaply commercially available. Expanding this model into preferences pertaining to other domains, or even for other penis shape preferences, appears desirable.

As a first study using life-sized 3D models of erect penises to investigate preferences, some limitations exist. Models were not perfectly ecologically valid. They were blue to minimize racial skin-color cues. They were made with rigid, odorless plastic. They were a simplified dome-on-cylinder form rather than realistically shaped and textured. The male body was neither described nor portrayed. There were also limitations of self-report approaches. Men and women appear to have actually become less approving of one-time sexual partners since 2001 [81], which may affect the preferences that they are willing to report regarding such partners. Also, a significant minority (15 of 75) of women chose not to report a preference for penis size in short and long term partners, but did answer both of the recall questions. Perhaps these women did not have a clear preference, consistent with weak penis size preferences reported in some previous studies [54, 65]. This could be viewed as a strength, insofar as women did not feel compelled to answer in cases where they did not feel they had a strong enough basis to generate an answer.

Another limitation is sexual inexperience among some participants. Fifteen women in our sample indicated that they had never experienced sexual intercourse. This inexperience could underlie some of the size preferences observed. For example, women generally anticipate more pain with their first intercourse than they actually experience [82], so they may show risk-averse penis size preferences (for shorter length and thinner circumference than they may prefer with experience). Less experienced women may also be less accurate in their size estimates. However, a follow-up analysis showed that having had sexual intercourse (yes or no) did not predict penis size preferences, arguing against this possibility. A related limitation is that the experimental protocol necessarily limited the sample size, and these women were recruited largely near a college campus. There may be other biases in the sample related to the recruitment method and sample size that were not identified.

There are several implications of these data for males interested in long-term female partners. Males with a larger penis may be at an advantage when pursuing short-term female partners. Also, this study provides the first data on the accuracy of women’s penis size judgments. Furthermore, women tended to slightly underestimate the length of penis models after a recall delay. Women may misremember specific partners penis attributes as smaller than they really are. This may exacerbate men’s anxieties about their penis size. Men dissatisfied with their penis size have historically benefitted more from counseling than from surgically increasing their penis size [83]. This may help explain why most men seeking surgical interventions for enlarging what they perceive to be a small penis actually have a penis that falls within a normal range [16]. Finally, 3D printing allows greater flexibility and complexity in stimuli and highly accurate replications. This first use of 3D stimuli to assess preferences is promising. Increasing print resolution and animation will broaden the research applications with haptic stimuli.
